# Mental Health, Spirituality, and Flourishing in New Medical Residents

**DOI:** 10.3390/jcm14207147

**Published:** 2025-10-10

**Authors:** Manuel Martínez-Sellés, Tyler J. VanderWeele

**Affiliations:** 1 Cardiology Department, Hospital General Universitario Gregorio Marañón, Instituto de Investigación Sanitaria Gregorio Marañón, CIBERCV, Calle Doctor Esquerdo, 46, 28007 Madrid, Spain; 2 School of Biomedical Sciences and Health Universidad Europea, School of Medicine, Universidad Complutense, College of Physicians, 28040 Madrid, Spain; 3 Human Flourishing Program and T. H. Chan School of Public Health, Harvard University, Cambridge, MA 02138, USA; tvanderw@hsph.harvard.edu

**Keywords:** depression, religion, psychology, medical profession

## Abstract

**Background/Objectives**: Depression is common in young doctors. Religion/spirituality, vocation, and family might protect against it. The objective of this study was to evaluate mental health, spirituality, and flourishing (a multi-dimensional assessment of well-being) in new medical residents. We also evaluated the associations of baseline characteristics with flourishing. **Methods**: We conducted a cross-sectional survey in 743 new medical residents who responded to (1) a questionnaire addressing mental health, vocation, spirituality, family, and religion and (2) the “Secure Flourish Index”, which assesses happiness/life satisfaction, physical/mental health, meaning/purpose, character/virtue, social relationships, and financial/material stability. **Results**: The mean age was 25.7 ± 4.0, and 526 (70.8%) were females, 157 (21.1%) had felt depressed, and 22 (3.0%) had suicidal ideation. The mean values for assessments of vocation and family were very high (>9.2), while religion and spirituality had intermediate values (5.2–6.5). Participants with depression or suicidal ideation had lower assessments in all four of the aforementioned domains, although differences were only significant for depression–religion (*p* = 0.03). The average “Secure Flourish Index” was 8.2 ± 0.9. There were lower levels of flourishing in males and in those with depression or suicidal ideation, with *p*-values < 0.001. Flourishing had a correlation with vocation and family (with r-values of 0.3–0.4) and with spirituality and religion (with r-values of 0.25–0.27), with *p*-values ≤ 0.001. **Conclusions**: Resident well-being is critical for physicians and healthcare quality. Spirituality and religion may serve as a protective resource, enhancing flourishing. Our study shows that new medical residents seem to have high value assessments for vocation and family importance and intermediate values for religion and spirituality. The mean flourishing scores were high, but were lower in males and in those with depression. Flourishing seems to be correlated with vocation, family, spirituality, and religion, but these associations need to be confirmed with validated screening tools.

## 1. Introduction

Previous studies show that depression is common in young doctors, and residency might be a challenge, where personal/family and professional roles can come into conflict [1]. Moreover, the transition from medical school to residency can be a critical period, with fears regarding stressful situations and new responsibilities. Medical residency might be a highly stressful period, with a high workload and long hours, sleep deprivation, financial strain, and increasing patient responsibilities. Stress and emotional exhaustion seem to be prevalent issues during medical residency and are associated with anxiety and depression, potentially leading to medical errors. In fact, high frequencies of depression and suicidal ideation have been documented in medical students [2,3], and burnout is more prevalent in residents and early-career physicians than among their older peers [4]. In addition, work–life balance is frequently seen as a major challenge, and few consider the option of starting a family during residency [5,6].

Flourishing indices are inversely related to depression metrics [7]. In fact, there has been an increasing interest in assessing multi-dimensional flourishing or complete human well-being in various populations [8,9,10,11,12]. Human flourishing can be defined as a state of overall, holistic well-being and optimal functioning in an individual’s life, encompassing health status, personal fulfillment, and positive social functioning. Flourishing has previously been assessed as performing or being well in five broad domains: (i) happiness and life satisfaction; (ii) health (both mental and physical); (iii) purpose; (iv) virtue; and (v) close social relationships. Like resilience, flourishing is inversely associated with depression, and this inverse association is particularly strong in the case of flourishing. Flourishing can be evaluated with standardized questions regarding multi-dimensional and complete well-being. As some of the factors measured to evaluate human flourishing can be associated with medical practice (for instance, happiness, health, meaning, character, relationships, and financial resources) [11], it makes sense to evaluate flourishing in medical residents. Moreover, flourishing assessments include mental and physical health, purpose, engagement, and relationships. All these factors are extremely important in medical practice. In the context of young physicians, flourishing could be a marker of professional growth, despite the stresses of the medical field. However, physicians, and particularly young doctors, have been infrequently studied, and studies regarding flourishing, mental health, and spirituality in medical residents are scarce. Spirituality might be of particular interest, as doctors’ satisfaction/happiness has been reported to be associated with spirituality and religion. The possible correlation between religion/spirituality and flourishing is relevant, as some studies suggest that both factors could work as shields against depression.

The objective of this study was to evaluate mental health, vocation, spirituality, family importance, and flourishing in new medical residents. We also assessed the associations of baseline characteristics with flourishing and the independent association of these variables with depression.

## 2. Materials and Methods

The present study was based on a cross-sectional descriptive survey performed in new medical residents. This questionnaire was also proposed to recently retired/about-to-retire physicians and their partners, but those data are not included in the current manuscript. Our participants were new residents from various medical specialties in the region of Madrid, Spain. The inclusion criterion was inscription for the first time in the College of Physicians to start residency training in 2024. This study was carried out in April and May 2024, as those were the months when young physicians in Spain started their medical residency. On registration day, all new members were asked to fill out, at the discretion of the respondent, a survey (Appendix A) in paper format or Google Forms. The questionnaire included the following.

(1) Specific Questions from the College of Physicians

All questions were selected after reviewing the literature [13,14,15,16,17,18,19,20] and included age, sex, marital status, information regarding medical profession (physicians as first-degree relatives and wanting their children to practice medicine), mental health (depression and suicidal ideation in the previous year), and questions regarding vocation, spirituality, family, and religion.

(2) The Secure Flourish Index

As noted above, human flourishing measures self-perceptions in five areas: happiness and life satisfaction, physical and mental health, meaning and purpose, character and virtue, and close social relationships. The Human Flourishing Program has developed a measurement approach to human flourishing, based around these five central domains. The flourishing index measure is copyrighted under a Creative Commons License (CC-BY-NC 4.0) and can be used without permission for non-commercial purposes if proper citation is given. The “Flourish Index” consists of two questions or items from each of five domains. The “Secure Flourish Index” also includes two additional questions on financial and material stability. Each of the questions is assessed on a scale of 0–10 [11]. Previous data have shown the Secure Flourish Index’s consistency, validity, and reliability [21].

The data were collected, organized, and cleaned. The Consensus-Based Checklist for Reporting Survey Studies (CROSS) was used [22].

### 2.1. Statistical Analyses

Quantitative variables were expressed as means with standard deviations (SDs). Qualitative variables were expressed as frequencies and percentages. Univariate analysis of data comparisons between two groups was performed using the unpaired t-test for normally distributed continuous variables. The equality of variances was assessed with Levene’s test. The lowest *p*-value for Levene’s test was 0.08, and the highest was 0.59. The Welch correction was not applied. The Pearson correlation coefficient (r) was used to test linear correlations between quantitative variables. Several stratifications with different cutoff points have been published to interpret r-values, and all have limitations [23,24]. We used the following cutoffs: 0–0.29 for a weak relationship, 0.3–0.7 for a moderate relationship, and >0.7 for a strong relationship [25].

Adjusted odds ratios were computed to assess associations with depression using logistic regression analysis. Stepwise logistic regression analysis was performed, which included variables that returned a *p*-value of < 0.1 in the univariate analysis.

All statistical analyses were performed using PASW Statistics for Windows, version 26.0 (SPSS Inc., Chicago, IL, USA).

### 2.2. Ethics and Informed Consent

Our study was based on a voluntary survey that was only completed by new medical residents of the Madrid College of Physicians who wanted to do so. The questionnaire was anonymous and included informed consent. For these types of surveys that are not administered in hospitals, the Spanish legislation does not require Institutional Review Board approval. The survey was approved by the Directive Board and the Ethics and Deontological Commission of the Madrid College of Physicians, which confirmed compliance with national and international guidelines. This study was conducted in accordance with the World Medical Association Declaration of Helsinki on Ethical Principles for Medical Research Involving Human Subjects, the Council of Europe Convention on Human Rights and Biomedicine (1997), and the Additional Protocol to the Convention on Human Rights and Biomedicine, in relation to Biomedical Research (2005). Data were collected, stored, and processed in encrypted/pseudo-anonymized forms. We also took into account the considerations contained in the International Ethical Guidelines for Health-Related Research Involving Human Subjects, developed by the Council for International Organizations of Medical Sciences in collaboration with the World Health Organization.

## 3. Results

A total of 1043 new medical residents were invited to complete the questionnaire. Of these, 745 responded, and after excluding 2 incomplete surveys, 743 valid responses were obtained, yielding a 71.2% response rate.

### 3.1. Specific Questions from the College of Physicians

The mean age was 25.7 ± 4.0 years, and 526 (70.8%) were females. The vast majority were single (713–96.0%) and would welcome their children practicing medicine (697–93.8%), and 211 (28.4%) had at least a parent or sibling physician. In the previous year, 157 (21.1%) had felt depressed, and 22 (3.0%) had suicidal ideation. The answers to quantitative questions rated from 0 (strongly disagree) to 10 (strongly agree) are shown in Table 1. The mean values for “vocation to care for and treat sick people” and “family is important” were very high (>9.2), while the assessment for identifying as a “religious person” or “spiritual person” had intermediate values (5.2–6.5). Participants with depression or suicidal ideation had lower assessments in all four items, although differences were only significant for depression–lower religious identification.

### 3.2. “Secure Flourish Index” and Associations with Baseline Characteristics

The mean responses to the “Secure Flourish Index” questions are provided in Table 2. With the exception of the last two questions regarding financial stability, all answers in the other domains (happiness, health, meaning, character, and relationships) had mean values of >8 points. Table 3 shows the average “Flourish Index” and “Secure Flourish Index” according to the baseline profile. We found lower levels of flourishing in males and in those with depression or suicidal ideation. Table 4 depicts the correlations of “Flourish Index” and “Secure Flourish Index” with vocation, family, spirituality, and religion. There was a moderate correlation with vocation and family (with r-values between 0.3 and 0.4) and a weak correlation with spirituality and religion (with r-values between 0.25 and 0.27).

### 3.3. Variables Independently Associated with Depression

Multivariate analyses showed that the “Flourish Index” and “Secure Flourish Index” were the only variables independently associated with the presence of depression in the last year (with odds ratios of 0.33–0.51 and 0.35–0.55, respectively, and both *p*-values < 0.001).

## 4. Discussion

In our sample of new medical residents, the values for vocation and family importance were very high, and the results were intermediate for religion and spirituality. Flourishing results were high and had a moderate correlation with vocation and family. The “Flourish Index” and “Secure Flourish Index” were the only variables independently associated with depression.

Our study’s findings regarding the high prevalence of depression and suicidal ideation point to the need for interventions to promote emotional health and resilience in young physicians [26]. A recent review found a higher depression prevalence in medical residents (8–93%) than in experienced physicians (5–67%) [27]. Even though the use of different diagnostic criteria and methodologies makes comparisons difficult, this prevalence appears to be increasing [28]. In addition, the prevalence of suicidal ideation and previously attempted suicide seems to be about 10% [29,30]. Addressing these high prevalences among medical residents requires a multifaceted approach targeting both individual and systemic factors. Some strategies might include reducing work hours, enhancing schedule flexibility, parental leave, and childcare policies, and decreasing barriers to mental health diagnosis and treatment [31]. Routine screening for depression may also be considered to help identify and target interventions for those with depression or with a high risk of developing it [32].

We also explored the interrelationships between mental health, religion, spirituality, and flourishing. Our data suggest that religion and spirituality are associated with overall well-being and may be related to professional growth. These findings are in accordance with previous evidence that religious participation is associated with well-being [33], as we found an inverse association of depression with religion; however, our results should not be overly interpreted, as they were obtained from cross-sectional data and cannot be used to assess causality. In any case, other longitudinal studies have suggested causal effects [34,35].

Flourishing measures included mental and physical health, purpose, engagement, and relationships. In the context of medical residents, flourishing can manifest as a sense of professional fulfillment, personal growth, and resilience. In the present sample, all individual flourishing items had mean values of ≥ 8.2, except the two questions that assessed financial and material stability (5.9 and 6.2, respectively). Our results show values higher than most international benchmarks [36,37], benchmarks conducted in other professions in Spain [38], and medical residents in other countries [39]. For instance, compared with other groups, as factory workers [36], medical residents in Spain seem to have better results. Moreover, some young populations might have low well-being scores [40]. The relatively low mean age of our participants (26 years) is relevant, as older doctors are usually happier than younger physicians [41]. The reasons behind the high well-being scores we found are unknown, but physicians in Spain are happier and more satisfied than other professionals with specialized education, such as lawyers or accountants [42]. Doctors’ satisfaction/happiness has been reported to be associated with meaning, love/relationships/family, and religion [43,44]. The fact that flourishing in females is higher than in males has been described [45,46] and is particularly relevant in our sample, as 71% of our new medical residents were females. A previous study that assessed students of medical sciences also reported a higher score of flourishing in females compared with males [47], but the reasons that underlie these differences are unknown. In fact, recent findings show that in the general population, the opposite seems to happen, with higher mean scores in men than in women [48].

We found a mild correlation between religion/spirituality and flourishing. New medical residents often experience high levels of stress, anxiety, and depression. Religion and spirituality could have the potential to foster resilience and flourishing, decreasing the risk of mental health issues by offering solace and perspective during the challenging time of medical residency. The finding that religion/spirituality is associated with the mental health of new medical residents is relevant, particularly if we take into consideration that over one-fifth of new residents had depression in the previous year. Supporting residents’ spiritual and global well-being could be seen as part of a holistic approach to their development. Residency programs might consider incorporating spirituality-oriented interventions and, when appropriate, facilitate religious practice, to help medical residents cope with the demands of their training. Core curricula for religiosity/spirituality in clinical practice during residency programs have been proposed, including topics such as religiosity/spirituality and mental health, taking into consideration a spiritual history/case formulation, main local traditions, differential diagnosis between spiritual experiences and mental disorders, and religiosity/spirituality integration in the approach to treatment [49]. Other proposed strategies include workshops regarding communicating bad news, lectures regarding various aspects of religious/spiritual practices and their implications on medicine, medical literature reviews relating to spirituality/religion and health, end-of-life lessons, interactions with pastoral care leaders, and ward rounds with faculty members regarding spiritual dimensions and faith-based resources [50]. Interventions for spiritual care in healthcare students and staff have focused on understanding spirituality in educational and practice settings [51]. Religious/spiritual coping, generally understood as the use of cognitive and behavioral strategies based on an individual’s beliefs and values to deal with stress, might benefit medical residents’ well-being [52]. In fact, better spiritual well-being of residents has been associated with a greater sense of work accomplishment, overall self-rated health, and decreased depressive symptoms [53]. Previous results also suggest that burnout emerges from dynamic interactions between systemic, institutional, and individual factors and may benefit from multipronged interventions [54]. Supporting physicians’ spiritual well-being as part of a holistic approach might increase their flourishing levels.

In any case, the highest correlations with flourishing were seen for the variables of vocation and family importance. Unfortunately, it is unclear how to improve these areas during medical residency. Vocation, described by Berg and Sadler [55] as serving as the spiritual/religious foundation for human flourishing, could also be the setting for flourishing in medical practice. However, some medical residents seem to reject the idea of medicine as a vocation, an idea entailing a blending of professional and personal moral identities, which is potentially exploitative and incompatible with the true source of meaning found outside of work [56]. As medical residents’ beliefs about work are personal, the sacrifices trainees make can be seen as worthwhile or unacceptable. Our data suggest that a strong commitment to patients might protect young physicians. Family is also essential in human flourishing; in our families, we learn to trust, cooperate, and self-restrain [57]. Among adolescents, greater family connection is associated with a higher prevalence of flourishing [58], and childhood family connection is associated with flourishing in adults [59]. Reconciling professional success with a fulfilling and satisfying personal and familial life is possible. There is arguably a need to remind residents that our work should not be the only priority in our lives [1]. Moreover, new strategies such as professional coaching might have the potential to improve physician well-being and might help to achieve an adequate work–family balance [60].

### 4.1. Limitations

This study had some limitations that warrant consideration. The sample included only new medical residents in Madrid, Spain, and the results might not be generalizable to other settings, particularly the items regarding financial and material stability, as socioeconomic situations and young physicians’ salaries might differ from other countries. Furthermore, our sample was predominantly female (71%), although these data are in accordance with the increasing proportion of women in the medical profession in Spain. In addition, we relied on self-report data, and there was, thus, a risk of misreporting depression. The first part of the questionnaire (specific questions from the College of Physicians) has not been validated. This self-designed few-minute questionnaire was created to increase the response rate, but it might underestimate or misclassify mental health outcomes. Due to this reason, data regarding depression and religion/spirituality should be regarded as hypothesis-generating. The second part (flourishing) involves a scale of multidimensional well-being with a validated Spanish version [61,62]. However, the high flourishing scores we found could be, in part, due to selection bias, as individuals who decided not to take the survey might have had lower flourishing results. We only asked about religiosity and have no data regarding specific religions. Nevertheless, most adults in Spain self-describe as Catholics, although the percentage is declining. Recent data showed that about 55% of Spaniards identified as Catholic and less than 5% as followers of other faiths (including Islam, Protestant Christianity, Judaism, Buddhism, and Hinduism) [63].

The key strengths of our study are the use of a questionnaire that included questions regarding mental health, spirituality, and flourishing, the large sample size, and the fact that it focused on a group (young physicians) where data are scarce.

### 4.2. Future Lines of Research

Validating and expanding flourishing as an outcome metric should probably be a priority in this field. Our study and previous data suggest that human flourishing is a valid, holistic indicator of medical resident well-being. Multi-center, large-scale longitudinal studies to validate flourishing as a standard outcome across different medical specialties are essential, as these data are needed to evaluate measures to improve residents’ well-being (e.g., mindfulness training, promoting spirituality, and structural changes). Some studies have suggested a benefit of mindfulness; in addition, spirituality in trainees is linked to resilience, empathy, and meaningful patient relationships. However, the real impact of these measures on flourishing over time is largely unknown. Validated baseline measures of depression and flourishing are essential to study the role of structural changes and formal well-being programs that could include reflective rounds and spiritual care training in depression prevention and flourishing promotion. Moreover, academic and clinical institutions should develop and test curricula that foster spiritual awareness and evaluate their long-term effects on medical residents’ resilience. A global research approach—combining validated metrics, educational innovation, organizational change, and personalized strategies—could help to clarify what truly works for whom, when, and why.

## 5. Conclusions

Resident well-being is critical, not only for the health of young physicians but for patient safety, empathy, and healthcare quality. Spirituality and religion, when supported and integrated meaningfully, may serve as a protective resource, enhancing resilience and flourishing. Our study shows that new medical residents seem to have high value assessments for vocation and family importance and intermediate values for religion and spirituality. The mean flourishing scores were high, but were lower in males and in those with depression. The flourishing score seems to be correlated with vocation, family, spirituality, and religion, but these associations need to be confirmed with validated screening tools and multifaceted research approaches.

## Figures and Tables

**Table 1:** Means and standard deviations of quantitative questions from the College of Physicians, rated from 0 (strongly disagree) to 10 (strongly agree) in (i) all participants and (ii) according to the presence of depression/suicidal ideation in the last year.

	All	Depression				Suicidal Ideation			
		Yes	No	*p*	Cohen’s d	Yes	No	*p*	Cohen’s d
Vocation	9.2 ± 1.1	9.1 ± 1.2	9.3 ± 1.1	0.06	1.1	9.1 ± 1.3	9.2 ± 1.1	0.43	1.1
Family	9.7 ± 0.8	9.5 ± 1.1	9.7 ± 0.7	0.05	0.8	8.9 ± 1.9	9.7 ± 0.7	0.07	0.8
Religion	5.2 ± 3.7	4.6 ± 3.7	5.3 ± 3.7	0.03	3.7	4.8 ± 3.9	5.2 ± 3.7	0.62	3.7
Spirituality	6.5 ± 2.9	6.2 ± 3.0	6.5 ± 2.8	0.24	2.8	6.1 ± 3.7	6.5 ± 2.8	0.58	2.9

**Table 2:** Means and standard deviations of “Secure Flourish Index”.

Flourishing Questions	
1. Overall, how satisfied are you with life as a whole these days?	8.7 ± 1.2
2. In general, how happy or unhappy do you usually feel?	8.3 ± 1.2
3. In general, how would you rate your physical health?	8.3 ± 1.4
4. How would you rate your overall mental health?	8.2 ± 1.3
5. Overall, to what extent do you feel the things you do in your life are worthwhile?	8.9 ± 1.3
6. I understand my purpose in life.	8.4 ± 1.6
7. I always act to promote good in all circumstances, even in difficult and challenging situations.	8.7 ± 1.1
8. I am always able to give up some happiness now for greater happiness later.	8.5 ± 1.3
9. I am content with my friendships and relationships.	9.1 ± 1.2
10. My relationships are as satisfying as I would want them to be.	8.6 ± 1.5
11. How often do you worry about being able to meet normal monthly living expenses?	5.9 ± 2.5
12. How often do you worry about safety, food, or housing?	6.2 ± 2.6
Average “Flourish Index” (questions 1–10)	8.6 ± 0.9
Average “Secure Flourish Index” (questions 1–12)	8.2 ± 0.9

**Table 3:** Means and standard deviations of (A) “Flourish Index” and (B) “Secure Flourish Index” according to baseline profile.

(A)			*p*	Cohen’s d
Sex: male vs. female	8.4 ± 0.9	8.7 ± 0.8	<0.001	0.9
Depression in the last year: yes vs. no	8.0 ± 1.0	8.7 ± 0.8	<0.001	0.8
Suicidal ideation in the last year: yes vs. no	7.2 ± 1.4	8.6 ± 0.8	<0.001	0.8
**(B)**				
Sex: male vs. female	8.0 ± 0.9	8.2 ± 0.8	0.001	0.9
Depression in the last year: yes vs. no	7.7 ± 1.0	8.3 ± 0.8	<0.001	0.8
Suicidal ideation in the last year: yes vs. no	6.8 ± 1.1	8.2 ± 0.8	<0.001	0.8

**Table 4:** Pearson correlation coefficient (r) values of (A) “Flourish Index” and (B) “Secure Flourish Index” with age, vocation, family, spirituality, and religion.

(A)	r	*p*
Age	0.05	0.20
Vocation	0.37	<0.001
Family	0.33	<0.001
Spirituality	0.25	<0.001
Religion	0.26	<0.001
**(B)**		
Age	0.03	0.41
Vocation	0.34	<0.001
Family	0.28	<0.001
Spirituality	0.27	<0.001
Religion	0.26	<0.001

## Data Availability

The dataset analyzed during the current study is kept in the College of Physicians of Madrid, Spain, and is available from the corresponding author upon reasonable request.

## Appendix A

**Specific Questions from the College of Physicians**

- Age_____
- Sex: Male___ Female___
- Marital status: Married___, Single ___, Widowed___, Separated___, Divorced___
- I am:

A physician and it is the first time I enroll in the College to start my practice ____

Recently retired/about to retire physician ___

Physician celebrating 50 years of membership___

Non-doctor, doctor’s partner____

- I have 1st degree relatives (parents, siblings or children) that are physicians: YES_NO
- If I have/had them, I would welcome my children and grandchildren becoming physicians: YES_ NO_
- In the last year have I felt depressed: YES_ NO_
- In the last year I have had suicidal ideation: YES_ NO_
- I consider that I have a vocation to care for and treat sick people____10—Strongly agree 0—Strongly disagree
- I consider myself a spiritual person____10—Strongly agree 0—Strongly disagree
- For me family is important____10—Strongly agree 0—Strongly disagree
- I consider myself a religious person____10—Strongly agree 0—Strongly disagree

**Flourishing Questions**

Domain 1: Happiness and Life Satisfaction.

1. Overall, how satisfied are you with life as a whole these days?

0 = Not Satisfied at All, 10 = Completely Satisfied

2. In general, how happy or unhappy do you usually feel?

0 = Extremely Unhappy, 10 = Extremely Happy

Domain 2: Mental and Physical Health.

3. In general, how would you rate your physical health?

0 = Poor, 10 = Excellent

4. How would you rate your overall mental health?

0 = Poor, 10 = Excellent

Domain 3: Meaning and Purpose.

5. Overall, to what extent do you feel the things you do in your life are worthwhile?

0 = Not at All Worthwhile, 10 = Completely Worthwhile

6. I understand my purpose in life.

0 = Strongly Disagree, 10 = Strongly Agree

Domain 4: Character and Virtue.

7. I always act to promote good in all circumstances, even in difficult and challenging situations.

0 = Not True of Me, 10 = Completely True of Me

8. I am always able to give up some happiness now for greater happiness later.

0 = Not True of Me, 10 = Completely True of Me

Domain 5: Close Social Relationships.

9. I am content with my friendships and relationships.

0 = Strongly Disagree, 10 = Strongly Agree

10. My relationships are as satisfying as I would want them to be.

0 = Strongly Disagree, 10 = Strongly Agree

Domain 6: Financial and Material Stability.

11. How often do you worry about being able to meet normal monthly living expenses?

0 = Worry All of the Time, 10 = Do Not Ever Worry

12. How often do you worry about safety, food, or housing?

0 = Worry All of the Time, 10 = Do Not Ever Worry
